# Genetic Aspects of Age-Related Macular Degeneration and Their Therapeutic Potential

**DOI:** 10.3390/ijms232113280

**Published:** 2022-10-31

**Authors:** Elisa Stradiotto, Davide Allegrini, Giovanni Fossati, Raffaele Raimondi, Tania Sorrentino, Domenico Tripepi, Gianmaria Barone, Antonio Inforzato, Mario R. Romano

**Affiliations:** 1 Department of Biomedical Sciences, Humanitas University, Via Rita Levi Montalcini 4, Pieve Emanuele, 20072 Milan, Italy; 2 Department of Ophthalmology, Eye Unit Humanitas Gavazzeni-Castelli, Via Mazzini 11, 24128 Bergamo, Italy; 3 IRCCS Humanitas Research Hospital, Via Manzoni 56, 20089 Rozzano-Milan, Italy

**Keywords:** AMD, polymorphism, genetic test, biomarkers, personalized treatments, complement therapy, gene therapy

## Abstract

Age-related macular degeneration (AMD) is a complex and multifactorial disease, resulting from the interaction of environmental and genetic factors. The continuous discovery of associations between genetic polymorphisms and AMD gives reason for the pivotal role attributed to the genetic component to its development. In that light, genetic tests and polygenic scores have been created to predict the risk of development and response to therapy. Still, none of them have yet been validated. Furthermore, there is no evidence from a clinical trial that the determination of the individual genetic structure can improve treatment outcomes. In this comprehensive review, we summarize the polymorphisms of the main pathogenetic ways involved in AMD development to identify which of them constitutes a potential therapeutic target. As complement overactivation plays a major role, the modulation of targeted complement proteins seems to be a promising therapeutic approach. Herein, we summarize the complement-modulating molecules now undergoing clinical trials, enlightening those in an advanced phase of trial. Gene therapy is a potential innovative one-time treatment, and its relevance is quickly evolving in the field of retinal diseases. We describe the state of the art of gene therapies now undergoing clinical trials both in the field of complement-suppressors and that of anti-VEGF.

## 1. Introduction

Age-related macular degeneration (AMD) has an estimated prevalence of 8% worldwide [1]. This condition mainly affects central vision; therefore, it is the major cause of visual acuity (VA)-lowering and blindness in the over-60-years-old population [2]. There are two types of advanced AMD, atrophic/dry AMD (85% of all AMD cases), where clinical signs are drusen and geographic atrophy (GA) in late disease, and exudative/wet AMD, in 15% of all AMD cases [3], where the invasion of abnormal choroidal neovascularization (CNV) occurs and subretinal fluid is present. The Age-related Eye Disease Study (AREDS) research group established a classification according to the clinical features of drusen, pigment abnormalities, GA, and CNV presence [4]. It is well known that family history and environmental factors influence susceptibility to AMD [5]. Over the last years, an increasing number of genetic mutations have been seen to impact AMD predisposition, thanks to genome-wide association studies (GWASs). In this review, we focus on the main genetic variants involved in the pathogenesis of AMD, especially considering mutations in pathways that seem to have a potential role as a therapeutic target. Accordingly, we present the molecules currently under clinical trial that exploit the knowledge acquired about the gene-variant effects of the main pathogenetic ways.

### Pathogenesis

**Figure 1:**
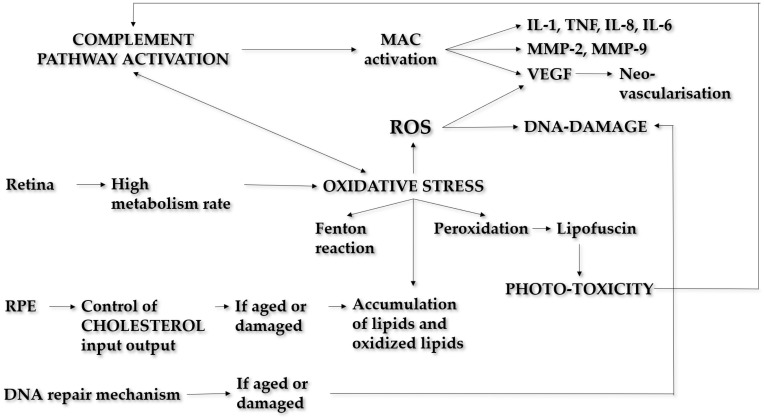
Summary of pathways involved in AMD pathogenesis and connections between them. Complement activation is the crucial mechanism. The last part of the cascade is the MAC activation, which releases various mediators of tissue damage: inflammatory molecules, tissue remodelling molecules, and VEGF. Oxidative stress is also considered a major risk factor in AMD as the retina is a high-rate-metabolism tissue. Through the Fenton reaction and peroxidation, tissue injury occurs, and the complement is triggered, thus perpetuating inflammation. ROS cause the production of misfolded proteins and DNA modification, particularly of mitochondrial DNA. If DNA-repairing mechanisms are less efficient because of age-related or genetic deficiencies, DNA changes and instability occur. RPE is the regulator of cholesterol input and output. When it becomes less performant, lipids and oxidized lipids aggregate, similarly to what happens in the atherosclerotic process. MAC: membrane attack complex; IL-1: interleukin 1; TNF: tumoral necrosis factor; IL-8: interleukin 8; IL-6: interleukin 6; MMP-2 matrix metalloproteinase 2; MMP-9 matrix metalloproteinase 9; ROS: reactive species of oxygen, RPE: retinal pigment epithelium.

Overactivation of the complement pathway is a fundamental contributor to AMD pathogenesis [6]. Any perturbations in homeostasis of the complement may lead to membrane attack complex (MAC) activation, the final step of the cascade. Sub-lytic MAC induces damage at the chorioretinal interface [7], elevating inflammatory mediators and molecules of tissue remodelling, increasing expression of adhesion molecules, activating inflammasome, and altering the balance between vascular endothelial growth factor (VEGF) and anti-angiogenic molecules [8,9]. The inflammatory response includes activation of both innate and adaptive immunity. Oxidative stress is a potential connection between the two systems, as lipid peroxidation products are capable of directly activating both M1 macrophages, whose recruitment is crucial to remove photoreceptor debris [10], and antigen-specific T cells [11]. (Figure 1).

The mechanisms of interaction between inflammation and oxidative stress are not fully clear but they are assumed to connect in many ways. Oxidative stress hinders RPE cells in controlling the activation of the complement system on their surface [12]. The macula is exposed to a high rate of oxidative stress because of its metabolism. Moreover, the photo-oxidative stress produced by processing light for vision is a unique example of an additional source of stress [13]. The RPE has a predominant antioxidative role: when it is less efficient, the tissue damage increases. As a consequence of cell stress, DNA changes occur (base modifications, DNA breaks, etc.), resulting in mutations and genome instability. The role of DNA repair in cell response to oxidative stress gives reason for the potential association between polymorphisms of DNA repair genes and risk of developing AMD [14,15]. ROS induce VEGF production in RPE cells, thus possibly causing CNV and AMD’s wet form [16], but oxidized cholesterol [17], inflammation, and modification of transcript events also seem to be related to angiogenetic events [18,19]. (Figure 1).

The RPE has a major role in cholesterol balance by regulating its input and output flow, and the uptake of oxidized low-density lipoprotein (LDL) by macrophages. When RPE becomes less performant, because it is damaged or aged, 7-ketocholesterol (7KCh) and other oxidized lipids aggregate. The increased number of lipid-saturated dysfunctional macrophages, called foam cells, is an important factor of local inflammation and a key event in the development of atherosclerosis, whose process resembles what happens in the retina during AMD development [20]. Furthermore, two main mechanisms of oxidation of cholesterol have been described in the retina: the Fenton reaction and photooxidation [21]. Through photooxidation and in the presence of a photosensitizer such as lipofuscin, cholesterol is converted into hydroperoxide intermediates that can further oxidize to 7kCh [22]. The products of the photo-oxidation of RPE lipofuscin trigger the complement system, contributing to chronic inflammation over time [23].

All those previously described pathways are linked together in many ways, most of which still need to be deeply understood.

As previously highlighted, the synergistic contribution of genetic and non-genetic factors leads to AMD insurgence and advancement (Table 1). Aging is probably the most important risk factor for AMD because it alters the molecular pathways involved in AMD pathogenesis, reducing the ability to respond and adapt to the impact of a multitude of exposures. Other environmental elements may strengthen such changes [24]. Smoking is the main influential modifiable risk factor. Long-time smokers have a significantly higher probability of developing disease than non-smokers of the same age [25]. Nutritional factors are thought to impact oxidative stress and inflammation. The AREDS research group found that the integration of high-dose antioxidants and zinc reduced the progression to late AMD, suggesting that dietary elements can contribute to the risk of AMD [26]. Additionally, other minor factors such as alcohol consumption [27], light exposure [28], and high blood pressure [29] have been associated with AMD. Several mechanisms may be influenced by environmental elements by modulating the activity or suppression of certain genes. Epigenetics, the study of modulations in gene expression that can be transferred over the generations without involving mutations in DNA [30], might better explain the environmental role and its deep connection to the genome [31]. Recent data in the literature suggest the involvement of epigenetics in the development of AMD through DNA methylation, changes in chromatin structure, histone acetylation, and inhibition of translation by micro-RNA (miRNA), a class of short single-stranded, non-coding RNAs, that prevents protein synthesis by binding messenger RNA [31]. miRNA can interfere with gene expression: many studies have suggested that dysregulated miRNAs may be involved in AMD pathology [32]. 

## 2. Methods

An online literature study (PubMed, Medline, Web of Knowledge, and Google Scholar) was performed to select relevant studies published until May 2022. Articles were searched using the key words: “gene”, “allele”, “polymorphism”, and “age macular degeneration”. The search included a total of 175 articles. All selected studies were critically analysed, and their bibliographies were meticulously evaluated to identify other pertinent articles. All clinical trials were evaluated on clinicaltrials.gov using the key words “retina biomarkers”, “retina genetic test”, “gene-therapy retina”, and “retina complement therapy”. Only clinical trials now active or completed but waiting for results to be published were selected.

## 3. Results

The results of our literature review are here reported in three sections to provide a comprehensive view from bench to bedside: AMD genomics, clinical applications, and future perspectives.

### 3.1. AMD Genomics 

The genetics of AMD is complex, involving 52 different variants in 34 genetic loci, according to the largest genome-wide association study ever conducted to date [33]. GWASs look for common low-penetrance genetic variants across many thousands of people. While genetic linkage studies are appropriate for monogenic traits, as their occurrence is determined by a single-gene high-penetrant mutation, GWASs are designed to disclose associations between a complex disorder and single-nucleotide polymorphisms (SNPs). Each SNP accounts only for a minimal part of the hereditary risk, but the additive effect of tens, hundreds, or thousands of them gives reason for a risk comparable to a single monogenic variant [34]. The first GWAS performed for AMD looked into Complement Factor H, followed by HTRA serine peptidase (ARMS2/HTRA1). Since then, subsequent GWASs for variants of candidate genes of the complement cascade, oxidative stress, lipid metabolism, DNA damage repairing, and neo-angiogenesis have been associated with AMD. In the present review, we discuss the main genetic variants, focusing on molecules currently considered potential therapeutic targets that are now undergoing clinical trial [33,34]. (Table 2).

#### 3.1.1. Immune Response and Complement Genes

##### Complement Factor H (CFH)

CFH activates the C3 cleavage and supports the degradation of C3 cleavage products. There is strong evidence that the rs1061170 CFH genetic variant tightly correlates to AMD [35,36] as it reduces the strength of the bond to heparan sulfate proteoglycans [58], causing lipoprotein deposition in Bruch’s membrane (BrM) and, consequently, drusen progression. The mutation affects CFH in a region that binds C-reactive protein (CRP), a major marker of inflammation [59]. Elevated serum levels of CRP have been reported to be associated with AMD [60]. CFH is also an innate defence against malondialdehyde (MDA), a molecule involved in lipid peroxidation. CFH H402 is a less efficient MDA-binding protein, thus weakening the bound of oxidized phospholipids. This mechanism further links oxidative, lipid, and complement dysregulation in AMD [61]. All these functional modifications affect the protein’s capacity as a complement regulator. Any reduced suppression of the complement way augments MAC levels and, together with oxidative stress, induces retinal cell damage and lowers the removal of debris, thus allowing the build-up of the waste products in drusen [62]. Sharma et al. [63] reported significantly lower serum CFH levels in the AMD Y402H variant of CFH compared to controls, showing its potential role as a biomarker for the disease. Variant rs1061170/Tyr402His is in strong linkage disequilibrium (LD) with rs570618. Another leading variant in *CFH* is rs10922109 [37]. Furthermore, rare CFH mutations have been disclosed and they are presumed to create meaningful disease phenotype features and earlier onset disease [64]: rs121913059 [38] shows increased risk regardless of the common variants. Additionally, other frameshift variants were identified, also independently of *CFH* Tyr402His [65,66,67]. CFHI62V polymorphism is a protective haplotype [65]. 

##### Complement-Factor-H-Related (CFHR)

Several data sources support the hypothesis that polymorphisms in extended *CFH* locus might have an impact on complement FH-related (CFHR) genes. The role of CFHR is still largely to be investigated but they are assumed to take part in complement regulation by competing with FH [68]. According to multiple studies, the deletion in those sequences exercises a protective role on AMD [69]. Altered FHR circulating levels have been reported in late AMD and to be associated with different genetic determinants [70]. 

#### Complement C3

The association between rs2230199 and rs1047286 in the C3 gene and AMD predisposition has been reported frequently [39,40]. Additionally, 71 rare missense variants were individuated, some of which were strongly associated with AMD [43,71]. 

##### Complement Factor B/Complement 2 (CFB/C2)

CFB is a genetic factor with a controlling role on the alternative complement, similarly to CFH. CFB rs641153 and rs415667 polymorphisms are associated with a protective effect for AMD and the presence of the C2 rs9332739 and rs547154 variants has been shown to reduce the progression to advanced AMD [41,42]. Subsequentially, variation in R32Q polymorphism has been reconducted with a clinical type of AMD dominated by small drusen and it seems to prevent the evolution to large drusen [72]. The minor allele of the rs116503776 loci in *C2/CFB* inversely correlates with disease evolution towards the final stage [37]. Globally, some rare alleles in CFB may cause reduced arousal of the alternative pathway and, therefore, they are presumed to perform a protective role against AMD onset.

##### Complement C9

A rare missense variant in C9 gene, rs34882957, is related to AMD [43]. Although the mechanism of its functional influence on the terminal part of the complement cascade has yet to be established [73], this is the first link between a component of the MAC and the development of AMD.

##### Complement Factor I (CFI)

The CFI gene produces a serine proteinase that is crucial to the complement control [74]. The rs2285714 carriers have been proven to be powerfully associated with AMD risk [44], and the mutation may serve as a biomarker [45], while rs10033900 may reduce the risk of AMD. An impaired CFI protein, similarly to highly penetrant variants of CFH, means insufficient complement-modulating activity, resulting in sustained complement attack.

##### Other Genetic Variants 

Many studies agree on the relationship in AMD between the inflammatory system, chemokines, and their corresponding ligands [75]. The chemokine receptors CX3CR1 and CCR-2 are displayed on macrophage surfaces after their activation and it has been suggested that they have a role in the angiogenic process for chemokine CCL2 through VEGF modulation [76]. 

SERPING1 encodes C1 inhibitor, a member of the serine proteinase inhibitor super-family, which mainly down-regulates the complement by preventing spontaneous activation. It is found in the neural retina, RPE, and choroidal tissue and it was seen to play a predisposing role to AMD in Caucasians [77].

Toll-like receptor 3 (TLR3) is a pivotal gene in the complex inflammatory machine that triggers programmed cell death in cells infected by virus thanks to their double-stranded RNA identification and is involved in the neo-angiogenetic process, as evidenced by its overexpression during the formation of CNV [78]. Variants causing a loss or reduction in those functions may play a role in the pathobiology of different subtypes of AMD [79]. 

The legacy of polymorphisms expressing complement mediators characterises the individual susceptibility to inflammatory response by changing the equilibrium between activation and suppression of the complement way. This inherited repertoire is identified as the “complotype”. Several lines of evidence support the idea that the complotype ranks a person at a certain level on a range from low to high intrinsic predisposition to complement activation [80]. These observations find practical relevance in the light of the new complement-modulating therapies that are now in the process of being developed: patients with a higher tendency for complement activation are more likely to take advantage of such therapeutic options [81].

#### 3.1.2. PLEKHA/ARMS2/HTRA-1

Together with CFH, PLEKHA/ARMS2/HTRA is the major frequent genetic risk factor for AMD. The rs10490924/A69S polymorphism, which surrounds two genes, ARMS2 and HTRA1, has been reproducibly observed as a strong genetic risk factor for AMD [46,47]. Although the mechanism causing the disease susceptibility is not fully clear, ARMS2 A69S polymorphism influences the anti-VEGF response in late-stage AMD, notably in the East Asian population [48], suggesting that the latter mutation may serve as a prognostic factor for the anti-VEGF response, especially in A-allele carriers or the AA genotype, whose detection may be an indication for early intervention [49]. Other reports identified the regulatory region HTRA1 rs11200638 [50,51]. HTRA1 encodes a heat shock serine protease that controls the cleavage of extra-cellular matrix (ECM) proteoglycans: its overexpression increases BrM membrane remodelling. As a result of the loss of structural architecture of the ECM, the choriocapillaris is more prone to neovascular invasion, as it occurs in wet AMD. Additionally, HTRA1 neutralises transforming growth factor-β (TGF-β), a protagonist in ECM apposition and neo-angiogenesis. From what emerges, it is appropriate to hypothesize that HTRA1′s destructive effects may help the CNV invasion [82]. These common variants are in perfect LD with rs3750486, the first uncovered SNP of the locus [37].

#### 3.1.3. Oxidative Stress Genes

The elevated metabolism of the retina exposes the tissue to an important load of oxidative stress [31], which is a mechanism of cellular damage determined by the loss of equilibrium between ROS creation and elimination. The extent of such damage depends on the efficiency of antioxidative mechanisms and the number of free radicals.

##### Manganese Superoxide Dismutase (MnSOD)

The manganese dismutase in the mitochondrial matrix is responsible for the protection of cells against generated ROS. There are four known functional polymorphism sites for MnSOD. Studies have found a significant correlation between the MnSOD gene Ala-9Val, Ile58Thr polymorphism, and wet AMD [52]. A more efficient manganese superoxide protein produces an excess of hydrogen peroxidase that may react with ferrous iron to form more cytotoxic hydroxy radicals, increasing the ROS load [53].

##### Iron Homeostasis Genes

Iron is a source of ROS generation through the Haber–Weiss and Fenton reactions, whose catalysation produces cellular injury. The amount of iron present is considerably higher in AMD-affected eyes than in healthy ones. Therefore, it is reasonable to speculate that iron storage may contribute to AMD [83]. Additionally, heme oxygenase-1 (HO-1), and heme oxygenase-2 (HO2), encoded by the HMOX 1 and 2 genes, respectively, are significant indicators of iron-induced oxidative stress. HMOX1 polymorphism rs2071747 was found to be associated with AMD evolution to the wet form, and HMOX2 polymorphism rs2270363 was associated with dry AMD [54].

#### 3.1.4. Lipid Metabolism Genes

Even though the true contribution of aberrant lipid regulation needs to be fully understood, the literature reports its involvement in AMD pathobiology, especially that of the high-density lipoprotein (HDL) cholesterol [84].

##### Apolipoproteins (Apo)

ApoE is involved in cholesterol transport and availability to cells by assisting the linkage between lipoproteins and LDL receptors. ApoE is located in photoreceptor segments, the retinal ganglion layer, and in BrM [85]. It is well documented that the ε2 allele of ApoE correlates with a higher risk of advanced AMD, while ε4 has a protective role for wet AMD [86,87]. Bakbak et al. [88] reported that mutated Apo ε4 shows consistently better VA after treatment with ranibizumab in exudative AMD. Apo E mutations could be used as screening for optimizing the therapeutic strategy in neovascular AMD.

##### ATP-Binding Cassette Family: ABCA1 and ABCA4

ABCA4 mutations are responsible for Stargardt disease, but different variants on that gene have a wide range of possible phenotypical manifestations, among which is included AMD. G1961E and D2177N have been found associated to be with AMD [89], but still there are contradictions among studies [33,90]. The ABCA4 loss of function provokes N-retinylidene PE complex accumulation, increasing the load of lipofuscin. Ultimately, cholesterol metabolism in the RPE cells becomes uncontrolled. ABCA1 is a membrane transporter that moves the overload of cholesterol from cells to lipid-poor apolipoprotein A-I (Apo A-I), the main component of HDLs. Its mutation causes a remarkable lack of HDL, cholesterol accumulation in macrophages, and increased cardiovascular risk. rs1883025 is associated with decreased risk of AMD development [55,91] while rs2740488 is associated with increased risk [33].

##### Hepatic Lipase (*LIPC*) 

*LIPC* is well represented in the retina and RPE, and it influences HDL cholesterol serum. The LIPC rs10468017 and rs493258 polymorphisms are considerably implicated in decreased risk of AMD [55], while rs2043085 is associated with advanced AMD [33]. The finding of a genetic variant in the HDL pathway could be taken into consideration to be used as a biomarker.

#### 3.1.5. Cell Survival Genes

##### DNA Damage Repairing

As previously said, the high rate of oxidative stress in the retina is a major source of DNA damage. In order to preserve DNA integrity, mechanisms for the DNA reparation are needed [92]. The basic mechanism is the nucleotide excision repair (NER) which is catalysed by four types of “uracil-DNA glycosylases” (UDGs): UNG, SMUG1 (single-strand selective monofunctional UDG), MBD4 (methyl binding domain 4 protein), and TDG (thymine/uracil mismatch DNA glycosylase). It has been suggested that polymorphisms in UDGs, SMUG1, and UNG2 genes may be implicated in AMD. The XPD gene encodes a DNA helicase implicated in transcription, NER, and apoptosis. Therefore, genetic variants of XPD could affect the efficiency of NER, cause DNA alteration, and contribute to the development of AMD [93]. Overall, further investigations are needed to identify genetic variants of proteins responsible for recognition and removal of oxidative DNA damage [14].

##### Nuclear Factor Kappa B (NF-κb)

NF-κb has a pivotal role within the inflammatory process, immune function, cell differentiation, proliferation, survival, and the autophagy program. Some polymorphisms are thought to be associated with AMD [94].

#### 3.1.6. Neovascularisation

##### Vascular Endothelial Growth Factor (VEGF)

VEGF strengthens the process of endothelial cell migration, proliferation, and vessel constitution. Furthermore, it has a pro-inflammatory function, inducing leukocyte migration; in turn, leukocytes stimulate VEGF production. Various SNPs, particularly rs943080, affect VEGF and VEGFR-2 levels, collaborating with the pathogenesis of neovascularization and impacting on the response to anti-VEGF treatment [56,57]

##### Tissue Inhibitor of Matrix Metalloproteinase 3 (TIMP3)

TIMP3 localises between RPE cells of the BrM where it binds MMPs delaying BrM matrix apposition, thus causing the thickening and reduction of the permeability of the membrane that leads to a deficiency in the circulation of nutrients between the choroid and the RPE [95]. Altered TIMP-3 is also responsible for Sorsby’s dystrophy, which shares some features with neovascular AMD. The loss-of-function mutations of TIMP-3 result in higher VEGF concentrations and aberrant vessel growth. Overall, TIMP-3 is known to inhibit CNV, but its upregulated levels are also associated with macular thickening and ensuing RPE atrophy [96].

#### 3.1.7. Extracellular Matrix Alterations

Fibuline 3 and (EFEMP1) and Fibuline 5 (FBLN5) [97] belong to the family of matrix glycoproteins containing a number of EGF-like sequences. EFEMP1 induces TIMP-1 and TIMP-3 release and neutralises several ECM metalloproteinases. EFEMP1 mutation is responsible for Doyne honeycomb retinal dystrophy, a maculopathy that has several common elements with AMD. In the AMD-affected macula, EFEMP1 lies misfolded between the RPE layer and drusen. Even if it is not an intrinsic component of drusen, it may lead to their deposition and the ensuing macular injury [98]. 

Fibuline 6 or Hemicentin (HMCN1) is an extracellular member of the immunoglobulin (Ig) superfamily. It resembles EFEMP1 as it also includes EGF-like repeats. HMCN1 is among the few genes which are held to be responsible for a Mendelian transmission of AMD.

### 3.2. Clinical Applications

The recent steps forward to the explanation of the genetic architecture of AMD give an account to the efforts made to develop genetic tests able to assess an individual’s risk of developing AMD and to predict treatment impact on clinical outcome.

#### 3.2.1. Association with Particular Clinical Subtypes of AMD

The CFH and ARMS2 high-risk SNPs are linked with progression toward GA and CNV, respectively [99,100]. 

Rare variants turned out to impact more incisively on phenotypic manifestation compared with non-carriers [101]. Rare *CFH* variants cause an earlier onset of late-stage disease, with more numerous, extramacular rather than macular, preferentially nasal to the optic disc, and crystalline or calcified drusen [102,103,104,105]. Moreover, a rare CFH missense variant, pArg1210Cys, apparently manifests with a peculiar cuticular phenotype [106]. Additionally, rare variants in the *CFH*, *CFI*, *C9*, and *C3* genes were found more often in GA than in wet AMD [101,105]. ABCA4 mutation is revealed with a granular pattern with punctate spots in the periphery, which resembles Stargardt disease [107]. An uncommon mutation in *TIMP3* (C1113G) gives precocious insurgence of disease and bilateral CNV [108]. Mutations in *FBLN5* have been correlated to cuticular drusen [109,110]. 

#### 3.2.2. Detection of Bio-Markers

All loci that proved to be AMD-related may be used as biomarkers to early diagnosis and to categorize the type of AMD in terms of clinical features, evolution, and response to therapy. 

Although the damage caused by the complement activation is localized, this does not exclude the consequences of its systemic impact [111]. The systemic complement activation contribution to disease pathogenesis remains controversial. A considerable number of studies tried to verify the relationship between genotype and altered plasma levels of complement protagonists in the AMD late stage. For instance, rare variants in *CFI* were reported to give reduced FI in serum [43,112]. Factors Bb and C5a were found to be independently associated with advanced AMD [113]. Although some evidence emerged, the results are discordant among different studies. Factor D and Factor I were found to be decreased in patients with AMD CFH Tyr402 mutation [114] and elevated in other studies [115]. Similar disputes were carried out for plasma levels of MAC components [7,116]. Even if a systemic detection of complement activation biomarkers is possible in AMD, they are thought to be a parallel phenomenon of what is going on at the eye level, rather than a consequence of the dysregulation process [117]. It has been noticed frequently that CRP plasma levels rise in AMD patients versus controls [60]: CRP possesses a CFH-binding site which enables it with a regulatory role, and, therefore, it can indirectly trigger the inflammatory response. Additionally, VEGF levels are usually exalted in individuals suffering from AMD [118]. Serum cholesterol levels may be associated with advanced AMD and with high-risk genotypes: LDL, Apo-A1 and HDLC levels have been described as elevated, whereas HDL and triglycerides were lowered compared to healthy patients [119,120]. Serum antibodies against cyclic nucleotide phosphodiesterase phosphatidylserine (PS) were tightly related to AMD at several degrees of the disease, observing a direct correlation between IgG/IgM arousal and the stage of AMD [121]. Serum Interferon γ-Inducible Protein 10 (IP-10) and eotaxin could constitute plasma biomarkers for precocious identification of AMD, for they were reported to increase in the earlier stages of AMD and enhanced in the GA more than in the late stage of the exudative form [122]. Levels of soluble FMS-like tyrosine kinase-1 (sFlt-1), which segregates VEGF-A that is extracellularly secreted, have been found to be hugely decreased in patients with wet AMD compared to those with early AMD and those without AMD [123].

Besides aspecific biomarkers (cholesterol, CRP, and VEGF), circulating autoantibodies, IP-10, eotaxin, and sFlt-1 are shown to be promising. Recently, long pentraxin (PTX3) has been suggested as a new biomarker: it softens the complement overreaction and prevents neo-angiogenesis in inflammatory eye disorders. It is also taken into consideration as a possible pharmacological target [124,125]. As robust biomarkers come out, the development of a tool that combines all various known indicators becomes plausible. Although a large number of possible biomarkers have been identified, none of them is exploited at any stage of disease diagnosis and management, either to identify high-risk individuals or address clinical management or predict treatment response [126]. A clinical trial (NCT04439708) is now ongoing to disclose biomarkers in the blood and aqueous humor in a population of AMD with occult CNV and analyse their relationship with the response to anti-VEGF treatment; another one (NCT05038371) aims to measure levels of connective tissue growth factor (CTGF) in the aqueous humor of patients with neovascular AMD and compare them to controls. Levels of VEGF will be measured as a positive control.

#### 3.2.3. Genetic Tests

AMD polymorphism may be useful to establish individual risk and to stratify patients according to their genetic risk factor for preventive and therapeutic studies. This is true particularly for the strongest and most common genetic variation of *CFH* and *ARMS/HTRA1* [82,127], which account for up to almost 70% of the heredity of AMD.

##### Prediction of Risk Profile

As the number of AMD-associated genetic variants increases, predictive models including all loci known until now take shape. The possibility to anticipate the risk of AMD onset would be a major step in the direction of personalised medicine, enabling preventive measures to be implemented in the case of high-risk assets and the development of therapies based on individual genetic structure. A polygenic risk score (PRS) is the sum of the chances to develop a given trait based on genetic susceptibility [128]. Each variant is weighted according to its size of impact on the disease [129]. The quantitative accuracy of PRS is measured using the area under the curve (AUC). The influence of the environment, the different weights of a genetic variant in different populations, and the dependence on the disease prevalence explain why a risk model with apparently adequate AUC may not be clinically reliable to assess true individual predisposition, as exemplified by Jakobsdottir et al. [130]. Despite the limitations, several models have been developed through the years [131,132,133,134] despite the fact that, in 2012, the American Academy of Ophthalmology advised against routinary genetic screening for multifactorial disorders such as AMD until reliable clinical trials could show specific preventive actions or treatment measures to be of benefit [135]. 

Currently available genetic tests use CFH and ARMS*/HTRA1* genetic variation. 

A comprehensive genetic test involving all 52 known common and rare gene variants and other rare variants was developed by Breuk et al. [136]. The assay was tested in 4740 participants; for each of them, the individual PRS was elaborated. Despite the observation of a higher PRS in advanced AMD in relation to healthy patients and early/intermediated AMD, an overlapping among groups persisted, so much so that it was not possible to completely discern the stage of disease only through PRS. The lack of evidence from clinical trials that use of genetic information could improve clinical outcomes, the difficulties in applying genetic testing to a clinical routine, and the inability to differentiate the severity of the stage weakens the use of the PRS for genetic testing. Overall, the costs and risks of routine genetic testing still overcome the advantages [137].

The purpose of the GARM II study, an ongoing clinical trial (NCT01115387), is to comprehend how genetic and environmental exposure (light, diet, and smoking) together contribute to susceptibility to develop the condition.

##### Response to Therapy

Genetic architecture affects more than just one’s risk of developing AMD; it may also impact the modality of response to the therapeutic intervention [138].

The CFH gene may influence treatment outcomes in patients with AMD [139], proving to be much less effective in Y402H AMD patients treated with anti-VEGF and suggesting that the mutation may be a useful predictor of treatment response; additionally, rs1065489 and rs800292 influence treatment outcomes after anti-VEGF [140]. Similarly to CFH, CFI rs2285714 is shown to respond worse to antiangiogenic treatment [141]. ARMS2 A69S is a prognostic factor for an anti-VEGF response in wet AMD in the East Asian population, but not in the Caucasian or Middle Eastern group [48]. HTRA1-rs11200638 SNPs suggested an improvement in VA for patients with the variated genotype [142]. The correlation between SNPs, such as *C3*, *CFB*, and *SERPING1,* and the anti-VEGF response to treatment in neovascular AMD has been investigated, with particular SNPs found to be associated with improved outcomes, compared to other SNPs giving a lack of treatment response. The C3 SNP rs2230199 demonstrates a better response, even if feeble, to anti-VEGF molecules. The VEGF SNPs rs3025000, rs833068, rs844069, and rs699946 record the most vigorous response to anti-VEGF treatment [143,144]. Recently, a study interestingly found that poor responders to ranicizumab demonstrate angiogenic and inflammatory processes enhanced by NF-κB signalling activation [145].

APOE ε4 allele carriers exhibit stronger VA after anti-VEGF treatment than APOE ε2 allele carriers [146].

Despite those observations, no clinical trials have yet shown that patient genotyping results in superior outcomes. A clinical trial (NCT03614481) is searching for susceptibility genes for AMD to retrieve genes that shape the AMD phenotype, especially regarding treatment response. 

Even though no treatment is currently approved for drusen accumulation, the knowledge of a high-risk profile could lead to the introduction of lifestyle changes (cholesterol control, smoke prevention, etc). Some clinical trials are now going in that direction. One of them (NCT05265624) aims to assess the impact of genetic testing for AMD on lifestyle behaviours as measured by systemic and ocular carotenoid status. Another clinical trial (NCT03024424) will evaluate the impact of genetic testing based on how it alters behaviours in order to investigate the utility of serum biomarker measurement in combination with genetic testing, the utility of genetic counselling in analysis of risk for AMD, and the impact of pre-symptomatic genetic testing for CNV. Besides the genetic test, pharmacogenetic testing could also assist in determining which genotypes are best suited to receive a particular kind of therapy. 

### 3.3. Future Perspectives

#### 3.3.1. Development of Personalized Treatments

Utilizing genotype information to define a high- risk population allows for customizing the approach and controlling the risk of developing the disease. As the most-explored mechanism of the disease by now is the alternative complement way, selecting a population with predominantly complement-driven disease is the first goal, although it is to define the best modality of administration (systemic or local) and the adequate section of the cascade to inhibit (amplification loop alone, C3, or C5 and beyond) [117]. Eculizumab’s inability to retard evolution towards atrophy suggests that systemic inhibition of the terminal part of complement way is a failing strategy [147]. According to the present evidence, the treatments should concentrate on the local complement overactivation, rather than on systemic modulation. Moreover, it seems that aiming at the complement final part of the cascade does not provide relevant therapeutic efficacy: efforts should be directed towards the amplification loop [148]. 

##### Complement

Currently, no American or European Medicines Agency approves any treatment against non-exudative AMD. The progression of knowledge about inflammatory genes implicated in AMD pathology justifies the complement suppressor molecules that are now under investigation as therapeutic options. Several clinical trials have been launched to verify the therapeutic role of the complement cascade inhibition and particularly the alternative way, whose implication on AMD pathology has been extensively studied.

Factor D is a protein of the alternative complement way that stimulates C3 convertase. Lampalizumab (Roche AG), a monoclonal antibody against Factor D administrated by intravitreal injection, gave encouraging results during the phase 2 MAHALO study but did not reduce GA growth rate versus placebo in the phase 3 trials, CHROMA and SPECTRI; the most complete study of GA performed until now [149]. One of the explanations of the failure of those trials stands on the incomplete inhibition of the inflammatory cascade, since only the alternative pathway was targeted, without causing any impact on the classical or lectin pathways. To the contrary, the blockage of C3 or C5 leads to full suppression of all pathways, so it is presumed to have better chance to delay GA progression. Danicopan, a complement Factor D inhibitor, is now under evaluation compared to a sham-controlled group in participants with GA secondary to AMD. (Table 3).

As previously discussed, CFH polymorphisms are strongly associated with AMD. Recombinant CFH molecules are assumed to reduce the expansion of disease in dry AMD caused by CFH gene loss of function [150]. The safety and tolerability of a single dose of intravitreally administered GEM103 (Gemini Therapeutics), a full-length recombinant CFH, is now being evaluated in the Phase 2a ReGAtta study in dry AMD (NCT04643886). This molecule shows activity profiles comparable to serum-derived CFH (sdCFH), presuming GEM103 equivalence in vitro to the original glycoprotein. These results encourage more research on GEM103 as a potential therapy for AMD [151]. Additionally, a novel peptidic complement inhibitor AMY-101 (Amyndas Pharmaceuticals, mini-FH) inhibits the splitting of native C3 into its cleavage products C3a and C3b and is now administrated in healthy male volunteers. 

The phase 2 GOLDEN study (NCT03815825) is now evaluating the ability of an antisense oligonucleotide (ASO) IONIS-FB-L_RX_ (Ionis Pharma, in partnership with Roche), administrated subcutaneously at multiple ascending doses every 4 weeks, on slowing GA lesion growth in AMD patients. This novel specific ASO targets the gene encoding for the human CFB. It was previously showed to reduce circulating levels of FB, thereby decreasing alternative pathway activity, in healthy volunteers [152]. Currently, the greatest hope for complement-based therapies is placed on Pegcetacoplan (APL-2, Apellis Pharmaceuticals). It is a cyclic peptide connected to a polyethylene glycol (PEG) polymer that captures C3 and C3b, impairs C3 cleavage, and prevents the opsonization which underlies the process of phagocytosis (Table 3). This C3 inhibitor, locally administrated via intravitreal injection, has proven to mitigate GA enlargement versus placebo treatment [153]. The FILLY study found that Pegcetacoplan treatment monthly resulted in a 29% of reduction of atrophic progression compared to sham controls, even after controlling for confounding factors [154]. To confirm APL-2 success, two phase 3 studies have completed enrolment (OAKS NCT03525613 and DERBY NCT03525600) and will define its efficacy and safety profile as a GA treatment.

Complement component 5 (C5) is the terminal common step of the complement cascade. C5 is cleaved into C5a and C5b fragments: C5a, together with C3a, is an anaphylatoxin; C5b gathers C6, C7, C8, and C9 to build MAC, the machine responsible for osmotic lysis and direct action against pathogens. Since C5 neutralisation theoretically preservers upstream activity, this method should apport the desired benefits due to complement inhibition reducing side effects. Eculizumab, a C5 antibody, seems to have no efficacy according to the COMPLETE study [147]. Similarly, LGF316 (tesidolumab) is an IgG1 antibody with affinity to C5 which blocks its splitting into C5a and C5b, whose presence is required for MAC deposition. That molecule was successfully tested by a 5 mg intravitreal injection administration in a phase 1 trial but failed at phase 2, showing no reduction in GA enlargement when compared to sham injections [155]. A trial arranging LFG316 (Novartis) together with the anti-properdin antibody CLG561 in GA AMD (NCT02515942) explores the impact of a double hit on the amplification loop and C5 activation. By now, the results have not been published. Previously, CLG561 (Novartis) failed in slowing the rate of GA expansion in a phase 2 monotherapy study [156] (Table 3).

Intravitreal administration of Avacincaptad pegol, a pegylated RNA aptamer which is another C5 agent, also known as Zimura (IVERIC bio), proved to be safe and effective in reducing GA growth in AMD eyes in the GATHER1 study by preventing the formation of C5a and C5b independently to the starting point of the pathway (alternate, classic, or lectin) [157]. Together with APL-2, this molecule is currently at the most advanced stage of study, as two phases 3 clinical trials are now evaluating its efficacy and safety in reducing GA progression (the GATHER2 Study, NCT04435366 and NCT02686658). Additionally, a phase 2 trial combining intravitreal injection of Zimura and Lucentis together in treatment of naïve subjects with neovascular AMD has been completed and results are being analysed (NCT03362190).

#### 3.3.2. Gene Therapy in AMD 

Gene therapy is an actively evolving approach in the field of inherited retinal diseases, where multiple trials have been launched and completed, leading to the FDA and (voretigene neparvovec) produced by Novartis and Spark Therapeutics Inc. to treat RPE65 deficiency [36]. Additionally, it is shown to be promising as an AMD potential therapy. Restoring the delicate network of connections of retinal tissue is an arduous task once atrophy occurs. The objective to be pursued is to stop photoreceptor degeneration by repairing the RPE layer before it is compromised [158]. Gene therapy aspires to the permanent and continuous expression of deficient-in-quantity or -in-quality proteins due to the underlying genetic defect. Secondarily, with gene therapy, only one injection could be resolutive for a lifetime. This is particularly relevant if applied to chronic conditions that need repeated treatments at frequent intervals [159]. The eye represents an extraordinary model for gene therapy because of its privileged position from an immunologic point of view that limits the possible reaction to the introduced genetic material and because of the blood–ocular barrier that limits systemic diffusion [160]. 

Cells normally express on their surface CD59, whose function is to inhibit MAC deposition. AAVCAGsCD59 (Janssen Research & Development), a gene therapy engineered for eyes, induces retinal cells to produce the soluble form of CD59 (sCD59) with the aim to obstruct the MAC accumulation in the macular retina, thus reducing the consequent damage. The point of this strategy lies in the observation that the choriocapillaris is uniquely sensitive to the deposition of MAC and that the choriocapillaris MAC deposition increases throughout life [161]. Multiple studies are now investigating safety after one injection of AAVCAGsCD59 in atrophic advanced AMD. HMR-1002 (NCT03585556) is a trial analysing the double dose of HMR59 in wet-AMD-affected eyes, while the HMR-1001 (NCT03144999) trial expects to deliver a low, mid, or high dose of HMR59 in dry AMD. Both trials will evaluate long-term follow-up safety and efficacy. GT005 (Gyroscope Therapeutics Limited) contains recombinant, non-replicating viral vectors derived from adeno-associated viruses (AAVs) containing the DNA-encoding CFI protein. It is now in phase 1 (FOCUS, NCT03846193) and phase 2 (HORIZON, NCT04566445, and EXPLORE, NCT04437368) of the evaluation of safety, dose-response, and efficacy after three doses of GT005 in one subretinal injection in individuals with GA where the disease is strongly related to genetic factors.

##### Anti-VEGF

The anti-VEGFs currently represent the gold-standard therapy for wet AMD, although their percentage of success may be improved, and, even when successful, the reduction in the quality of life resulting from a treatment so frequently repeated over time is a major cause of low compliance of patients. Therefore, novel AMD treatment approaches are needed. Numerous treatment options of gene therapy for neovascular AMD are in phase 1/2 clinical trials, usually requiring a single injection, which may be way for the development of a personalized, less invasive, and more clinically adaptable approach in wet AMD. (Table 4).

AAV2-sFLT01 (Genzyme, a Sanofi Company) is a single intravitreally delivered dose of a vector carrying sFLT-1, a VEGF-A inhibitor [162]. Another company developed a recombinant adeno-associated virus (rAAV) gene therapy, known as rAAV.sFlt-1 (NCT01024998), that only differs from the previously described product in the sFLT-1 fusion sequence binding the Fc region of IgG1 [160]. This is thought to prevent CNV formation through inducible over-expression of Eylea in wet AMD [163]. RGX-314 (Regenxbio, Inc) is a rAAV vector encoding the genetic material for the expression of a soluble anti-VEGF monoclonal antibody molecule resembling ranibizumab. The ongoing phase I/IIa trial explores the safety and efficacy of RGX-314 subretinal injection, which is shown to be well tolerated [164]. Several clinical trials are now underway to determine its efficacy as a potential single-administration treatment for wet AMD (NCT04514653, NCT04832724, NCT03999801, NCT05210803, and NCT04704921).

ADVM-022 (Adverum Biotechnologies) is a rAVV replication-deficient vector expressing aflibercept. Preclinical evaluation of ADVM-022 claims that a one-time administration may provide a long-term effective therapy for wet AMD [165]. A phase 1 clinical trial is now underway to explore its safety and effectiveness (NCT03748784). With only one exception, the aflibercept levels detected after ADVM-022 administration were within the therapeutic range [166].

The treatment 4D-150 (4D Molecular Therapeutics) is an AAV vector carrying aflibercept and sequences of miRNA that target VEGF-C and. The clinical trial currently running (NCT05197270) provides for a one-time administration in patients who have shown a clinical response to anti-VEGF and a 24-month follow up. RetinoStat (Oxford BioMedica), also known as OXB-201, is a lentiviral vector expressing endostatin and angiostatin [167] which was proven to be safe and well tolerated and provides reproducible, sustained transgene expression. A reduction in neovascular activity was obtained in 71% of cases, but in only one case did significant reduction in intraretinal/subretinal fluid occur [168]. The long-term safety is now under evaluation (NCT01678872). IBI302 (Innovent Biologics Co. Ltd.) is a decoy receptor structured with a VEGF suppressor sequence (containing both extracellular domain 2 of VEGFR1 and the extracellular domain 3 of VEGFR2) and a complement inhibitor fragment; these domains are linked together by the Fc region of Ig. IBI302 demonstrated in vitro neutralisation of endothelial cell proliferation and of the complement way [169]. The complement inhibition domain also includes a complement receptor binding site (CR1). CR1 supports the cleavage of both C4b and C3b. Furthermore, it promotes the decay activity against C3 convertases [170]. Wang et al. [171] claim that IBI302 could help to maintain the RPE integrity. A phase II study is now going (NCT04820452).

Additionally, an early phase I study (NCT05099094) is evaluating BD311 (Shanghai BDgene Co., Ltd.), an integration-deficient lentiviral vector (IDLV)-expressing VEGFA antibody effective in exudative AMD in a murine model [172]. 

Future phase 3 trial results will determine which of these therapies will be the next novel treatment of wet AMD.

## 4. Conclusions

This review traces the main pathogenetic pathways associated with the development of AMD known today, and their respective polymorphisms, to present an overview of the molecules currently ongoing clinical trials to validate their therapeutic efficacy. We highlight the importance of an individual assessment of genetic set-up to guide the therapeutic approach and particularly to discriminate those who would benefit from complement suppression from those whose disease is supported by other dysregulating mechanisms.

Some new treatments have shown promising results so far, both in the anti-VEGF activity and complement modulation fields. Progress has been made, especially on the latter one: it is now clearer which is the best part of the cascade to suppress and which route of administration to choose. In particular, two pharmaceutical companies are in advanced stage of developing complement-based drugs, APL-2 and Zimura, whose upcoming phase III trials will disclose the potential of complement modulation as a therapeutic option.

In the light of Luxturna approval for retinal diseases, we gave special attention to gene therapy as a potentially innovative, powerful, and longstanding therapeutic option. Several phase I and II trials are looking for gene-based therapy to induce constitutive cell production of anti-VEGF and complement products.

These two ways seem to play a pivotal role in the near future, but as other dysregulating mechanisms are discovered, different therapeutic options may be explored.

## Figures and Tables

**Table 1:** Environmental risk factors for AMD, divided into no modifiable and modifiable factors.

Environmental Factors	No Modifiable	Modifiable
	AGE	SMOKING
	EPIGENETICS DNA methylation Chromatine changes Histone acetylation miRNA	NUTRITION
		ALCOHOL
		LIGHT EXPOSURE
		HIGH BLOOD PRESSURE

**Table 2:** Complement component 3; CFB: complement factor B; C2: complement component 2; C9: complement component 9; CFI: complement factor I; MnSOD: manganese superoxide dismutase, HMOX1: heme oxygenase-1; HMOX2: heme oxygenase-2; Apo E: apolipoprotein E; ABCA 1: ATP-binding cassette 1; LIPIC: Hepatic Lipase; VEGF: Vascular Endothelial Growth Factor.

Pathway	Molecule	Polymorphism	Evidence
Immune response and complement genes	CFH	rs1061170, rs10922109, rs121913059	Fritsche et al., 2016 [33], Edwards et al., 2005 [35], Park et al., 2019 [36], Herrsterbeek et al., 2020 [37], Ferreira et al., 2009 [38]
	C3	rs2230199, rs1047286	Maller et al., 2007 [39], Thakkinstian et al., 2010 [40]
	CFB	rs641153, rs415667	Wang et al., 2013 [41], Thakkinstian et al., 2012 [42]
	C2	rs9332739, rs547154	Wang et al., 2013 [41], Thakkinstian et al., 2012 [42]
	C9	rs62358361, rs34882957	Fritsche et al., 2016 [33], Seddon et al., 2013 [43],
	CFI	rs2285714, rs10033900	Ven et al., 2013 [44], Wang et al., 2016 [45]
other	PLEKHA/ARMS2/HTRA-1	rs10490924, rs11200638	Kanda et al., 2007 [46], Mullins et al., 2019 [47], Zhang et al., 2021 [48], Liu et al., 2020 [49], DeWan et al., 2006 [50], Chen et al., 2009 [51]
Oxidative stress genes	MnSOD	Ala-9Val, Ile58Thr	Kimura et al., 2020 [52], Kowalski et al., 2010 [53]
	HMOX1 HMOX2	rs2071747, rs2270363	Synowiec et al., 2012 [54]
Lipid metabolism genes	ApoE	rs429358	Fritsche et al., 2016 [33]
	ABCA1	rs2740488	Fritsche et al., 2016 [33]
	LIPC	rs0468017, rs493258, rs2043085	Yu et al., 2011 [55], Fritsche et al., 2016 [33]
Neovascularisation genes	VEGF	rs943080	Zaho et al., 2013 [56], Balikova et al., 2019 [57]

**Table 3:** Summary of complement-modulating molecules undergoing clinical trials, gene-therapy-based and not.

Complement						
Antagonist	Therapeutic (Alt. Name)	Pharma Company	Treatment Type	Complement Target	Administration	Clinical Trials
	GEM 103	Gemini Therapeutics	full-length recombinant	CFH	Intravitreal injection	NCT04643886 phase II
	AMY 101	Amyndas Pharmaceuticals	peptidic complement inhibitor (mini-FH)	CFH		administrated in healthy male volunteers
	IONIS-FB-L_RX_,	Ionis Pharma	antisense oligonucleotide encoding CFB	CFB	Subcutaneous	NCT03815825 phase II
	Pegcetacoplan (APL-2)	Apellis Pharmaceuticals	cyclic PEG peptide	C3	Intravitreal injection	NCT03525613 NCT03525600 phase III
	LGF 316 + CLG561	Novartis	IgG1 antibody + anti-properdin antibody	C5 + properdine	Intravitreal injection	NCT02515942 (completed)
	ZIMURA (ARC1905)	IVERIC bio	pegylated RNA aptamer	C5	Intravitreal injection	NCT04435366 phase III
Gene-Therapy	AAVCAGsCD59 (HMR59)	Janssen Research & Development	AAV gene therapy	CD59	Single intravitreal injection	NCT03585556 NCT03144999 (completed)
	GT005	Gyroscope Therapeutics Limited	AAV gene therapy	CFI	Single subretinal injection	NCT03846193 phase I NCT04566445 NCT04437368 phase II

**Table 4:** Summary of anti-VEGF gene therapies now undergoing clinical trials.

Anti-Angiogenesis					
Gene Therapy	Therapeutic (Alt. Name)	Pharms Company	Gene Expression	Administration	Clinical Trials
	AAV2-sFLT01	Genzyme, a Sanofi Company (Modena, Italy)	sFLT01	Single intravitreal injection	NCT01024998 phase I
	RGX-314	Regenxbio, Inc. Rockville, MD, USA	monoclonal antibody fragment similar to ranizumab	Subretinal via transvitreal injection	NCT04514653, NCT04832724, NCT03999801, NCT05210803, NCT04704921
	ADVM-022	Adverum Biotechnologies (Redwood City, CA, USA)	coding sequence for aflibercept	Single intravitreal injection	NCT03748784 phase I
	4D-150	4D Molecular Therapeutics (Emeryville, CA, USA)	miRNA targeting VEGF-C and sequences encoding aflibercept	Single intravitreal injection	NCT05197270 phase I
	RetinoStat (OXB-201)	Oxford BioMedica (Oxford, UK)	endostatin and angiostatin	Intravitreal injection	NCT01678872 phase I
	IBI302	Innovent Biologics Co. Ltd. (Suzhou, China)	decoy receptor fusion protein	Intravitreal injection	NCT04820452 phase II
	BD311	Shanghai BDgene Co., Ltd. (Shanghai, China)	expressing VEGFA antibody	Intravitreal injection	NCT05099094 phase I

## Data Availability

Data available upon request.
